# Meta-analysis of dynamic contrast enhancement and diffusion-weighted MRI for differentiation of benign from malignant non-mass enhancement breast lesions

**DOI:** 10.3389/fonc.2024.1332783

**Published:** 2024-03-12

**Authors:** Jing Zhang, Longchao Li, Li Zhang, Xia Zhe, Min Tang, Xiaoyan Lei, Xiaoling Zhang

**Affiliations:** Department of Magnetic Resonance Imaging (MRI), Shaanxi Provincial People’s Hospital, Xi’an, Shaanxi, China

**Keywords:** non-mass enhancement lesions, meta-analysis, breast cancer, dynamic contrast enhancement, diffusion-weighted imaging

## Abstract

**Purpose:**

The objective of this study was to conduct a meta-analysis comparing the diagnostic efficacy of models based on diffusion-weighted imaging (DWI)-MRI, dynamic contrast enhancement (DCE)-MRI, and combination models (DCE and DWI) in distinguishing benign from malignant non-mass enhancement (NME) breast lesions.

**Materials and methods:**

PubMed, Embase, and Cochrane Library were searched, from inception to January 30, 2023, for studies that used DCE or DWI-MRI for the prediction of NME breast cancer patients. A bivariate random-effects model was used to calculate the meta-analytic sensitivity, specificity, and area under the curve (AUC) of the DCE, DWI, and combination models. Subgroup analysis and meta-regression analysis were performed to find the source of heterogeneity.

**Results:**

Of the 838 articles screened, 18 were eligible for analysis (13 on DCE, five on DWI, and four studies reporting the diagnostic accuracy of both DCE and DWI). The funnel plot showed no publication bias (*p* > 0.5). The pooled sensitivity and specificity and the AUC of the DCE, DWI, and combination models were 0.58, 0.72, and 0.70, respectively; 0.84, 0.69, and 0.84, respectively; and 0.88, 0.79, 0.90, respectively. The meta-analysis found no evidence of a threshold effect and significant heterogeneity among trials in terms of DCE sensitivity and specificity, as well as DWI specificity alone (I^2^ > 75%). The meta-regression revealed that different diagnostic criteria contributed to the DCE study’s heterogeneity (*p* < 0.05). Different reference criteria significantly influenced the heterogeneity of the DWI model (*p* < 0.05). Subgroup analysis revealed that clustered ring enhancement (CRE) had the highest pooled specificity (0.92) among other DCE features. The apparent diffusion coefficient (ADC) with a mean threshold <1.3 × 10^−3^ mm^2^/s had a slightly higher sensitivity of 0.86 compared to 0.82 with an ADC of ≥1.3 × 10^−3^ mm^2^/s.

**Conclusion:**

The combination model (DCE and DWI) outperformed DCE or DWI alone in identifying benign and malignant NME lesions. The DCE-CRE feature was the most specific test for ruling in NME cancers.

## Introduction

According to the Breast Imaging-Reporting and Data System (BI-RADS) MRI vocabulary, “non-mass enhancement (NME)” refers to distribution and internal enhancement that do not meet the requirements for a mass after injecting a contrast medium (1, 2). However, there is a lack of characteristic manifestations of breast NME lesions, and the overlap of various features of non-mass breast cancer (BCa) with benign breast lesions, like fibrocystic and inflammatory alterations, as well as focal adenosis, can be found in malignant lesions such as lobular carcinoma, diffuse invasive BCa, invasive ductal carcinoma, ductal carcinoma *in situ* (DCIS), and, on rare occasions, specific forms of BCa (3, 4).

The descriptors of BI-RADS for NME are limited to morphological enhancement and distribution patterns (5). It can be difficult to distinguish between benign and malignant NME using breast MRI for BI-RADS diagnosis (6). Because MRI-guided biopsies are rarely used, it is common to perform unnecessary or delayed surgery.

Currently, most institutions prefer dynamic contrast enhancement (DCE) MRI to diagnose NME. DCE can reveal morphological features (focal, linear, and segmental), enhancement characteristics (homogeneous, heterogeneous, clumped, and clustered ring enhancement), and kinetic patterns, which are valuable for distinguishing benign NME lesions from malignant NME tumors (6).

The internal enhancing characteristics of NME lesions provide less information about malignancy than do those of masses. NME is the most common presentation for DCIS and non-palpable invasive malignancies (7, 8), despite the prevalence of NME lesions on DCE-MRI being significantly lower than that of masses (76% versus 13%). High-risk lesions, benign disorders such as fibrocystic disease, and hormone alterations have also been connected to NME (9, 10). Consequently, NME cancers present a challenge for breast DCE-MRI.

Diffusion-weighted imaging (DWI) is one functional imaging technique that is gaining popularity as a means of increasing accuracy; it may also prove to be a useful tool for the diagnosis and treatment of individuals with NME lesions. DWI is becoming a routine technique for breast MRI, despite the fact that the apparent diffusion coefficient (ADC) value is not included as a classification indicator in the BI-RADS 2013. Several studies have demonstrated the effectiveness of DWI in the detection of NME lesions (11, 12). DWI with ADC mapping is now advised in conjunction with DCE-MRI within a clinical multiparametric (mp) MRI strategy (13–15). It significantly enhances specificity in distinguishing between benign and malignant breast tumors, avoiding unnecessary breast biopsies (11, 12, 16, 17).

Shao et al. investigated the diagnostic utility of the DCE in NME lesions approximately 10 years ago (18). Many studies have now demonstrated the efficacy of DWI in the diagnosis and treatment of NME breast cancers. They discovered that DWI provided similar but superior diagnostic information to DCE and that DWI could be combined with DCE to improve accuracy (11, 19). It is suspected that mpMRI and DWI are not as effective for distinguishing benign from malignant NME lesions (14). A detailed systematic review would be useful for analyzing the vast amount of information now available.

Therefore, we conducted a meta-analysis to examine the diagnostic efficacy of models based on the DWI, DCE, and combination models (DCE and DWI) in detecting NME cancer.

## Methods

The Preferred Reporting Items for Systematic Reviews and Meta-Analyses—Diagnostic Test Accuracy (PRISMA-DTA) statement (20) was followed in this meta-analysis. The Cochrane Collaboration Diagnostic Test Accuracy Working Group (21) was used to conduct this meta-analysis.

### Method for searching the literature

A comprehensive search for published research up to January 30, 2023, was conducted utilizing the PubMed, Embase, and Cochrane Library databases. The search terms were (“Nonmass” OR “Non-mass-like” OR “Non-Mass” OR “non-mass enhancement”) AND (“MR imaging” OR “MRI” OR “magnetic resonance imaging” OR “MR”) and “breast”. The search was limited to original studies written in English or Chinese and published on paper. Furthermore, the references of the included articles were manually searched.

### Study selection

Two researchers (L.**. and J.**. with 8 and 6 years of breast MRI experience, respectively) individually assessed the whole texts of any papers that might be qualified after screening the titles and abstracts of the papers that were retrieved. All disagreements regarding potential eligible papers were resolved by a third researcher (L.C.**. with 10 years of experience in breast MRI).

The inclusion criteria were as follows: a) patients: patients with NME lesions; b) index test: breast MRI was used as a diagnostic test for NME lesions; c) comparison: pathological and/or clinical follow-up results; d) outcomes: diagnostic accuracy for differentiating NME benign lesions from cancers; and e) study design: retrospective or prospective trials. We excluded studies without absolute numbers of true positive (TP), false positive (FP), true negative (TN), and false negative (FN) cases; and letters to the editor, case reports, conference abstracts, review articles, meta-analyses, and animal experiments.

### Data extraction

The data extracted from each study were the study characteristics (first author; publication year; country; study design; reference standard; sample size; cancer prevalence; percentage of DCIS in all cancers; subtype of malignant lesions; data source; diagnostic criteria; sample inclusion time; TP, FP, FN, and TN numbers), patient characteristics (age, gender, menopausal status, and number of total non-mass-like lesions), and imaging characteristics (MRI pulse sequences, b value, magnetic field strength, position of the patients, and slice thickness). If mean results were reported in the case of multiple reviewers, the results were used for the analyses; if not, the results of the more experienced reader were used (22). The two authors (L.**. and J.**.) extracted the data independently. Inconsistencies between the two researchers were re-evaluated by a third researcher (L.C.**.).

### Study quality assessment

Two authors (L.**. and J.**.) independently evaluated the quality of the included studies using the Quality Assessment of Diagnostic Accuracy Studies 2 (QUADAS-2) criteria (23). The final results were based on a consensus discussion.

### Statistical analysis

Statistical analyses were performed using R 4.1.2. Meta-analyses were carried out using the bivariate random-effects model (24). Pooled sensitivity and specificity were calculated using 2 × 2 contingency tables. Similarly, the areas under the curve (AUCs) of summary receiver operating characteristic (ROC) curves were estimated. Threshold effects were assessed by determining if the ROC curve followed a “shoulder-arm” distribution. The I^2^ statistic, which ranges from 0% to 100%, was used to assess the heterogeneity of study results. An I^2^ of more than 75% implies significant heterogeneity between groups (25).

Subgroup analyses and meta-regression were employed to investigate the effects of the heterogeneity-causing factors. Subgroup analyses were performed for studies with different DCE patterns of MR distribution: heterogeneous, clumped, and clustered ring enhancement (CRE), washout, plateau and plateau or washout time-signal intensity curve (TIC), and the different cut-offs of ADC values.

The meta-regression included the following covariates: a) predesign (prospective *vs.* retrospective), b) publication year (before *vs.* after 2013), c) diagnostic criteria [Internal enhancement pattern (IEP) *vs.* others; ADC values ≥1.3 × 10^−3^ or <1.3 × 10^−3^ mm^2^/s], d) MR magnet strength (1.5 T *vs.* 3.0 T), e) reference (histopathology *vs.* histopathology or follow-up), f) cancer prevalence ≥50% (yes *vs.* no), g) max b value ≥800 s/mm^2^ *vs.* < 800 s/mm^2^ or not reported, and h) race (Caucasian *vs.* Asian).

### Analysis of publication bias

Visual examination of funnel plots was used to evaluate potential publication bias (26). A *p*-value of less than 0.05 was considered statistically significant.

### Clinical utility

The clinical value was tested using a Fagan plot, which yielded the posttest probability (*p* post) of NME when the pretest probabilities (*p* pre, suspicion of NME) for the DCE, DWI alone, and combination models were computed (27).

## Results

### Literature search

**Figure 1:**
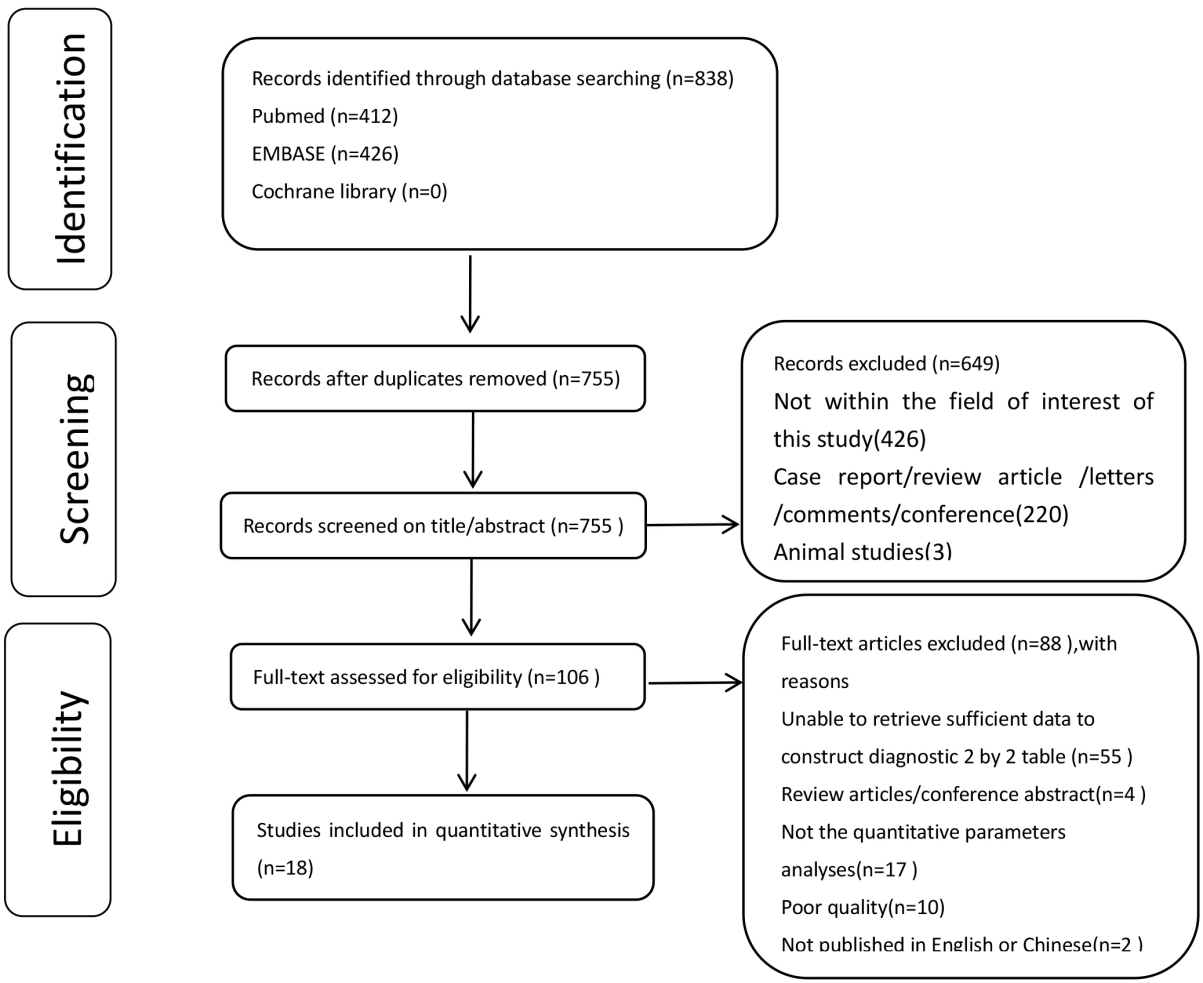
The flowchart of the study selection process.

The systematic literature search yielded 838 articles. After removing duplicates, 649 articles were excluded after title screening and abstract review. A total of 106 full-text articles were assessed for eligibility, of which 88 papers were excluded. Finally, 18 studies were included in the meta-analysis based on the diagnostic criteria since they reported sufficient quantitative data. **Figure 1** presents the flowchart of the study selection process. Among the 18 included studies, 13 reported on the diagnostic accuracy of DCE-MRI (5, 11, 13, 19, 28–36), nine evaluated DWI-MRI (11, 12, 14–17, 19, 30, 31), and four reported the diagnostic accuracy of both DCE and DWI (11, 12, 14, 19).

### Study and patient characteristics

**Table 1** shows the baseline characteristics of the included studies. The subjects were all female. Thirteen articles described 38 datasets that used the DCE sequence. Ten studies reported 10 datasets evaluating segmental features as the index test (5, 11, 13, 28–32, 34, 35). Four datasets assessed heterogeneous characteristics (5, 13, 32, 33), whereas six datasets used clumped characteristics (5, 11, 30–33). Five datasets employed the CRE characteristic (5, 29–31, 36), five datasets were analyzed based on the DCE washout TIC pattern (11, 13, 30–32), and five datasets were examined using the plateau TIC (11, 13, 30–32). Furthermore, six datasets used the washout/plateau TIC (11, 13, 19, 30–32), while four datasets used DCE combined with DWI as the index test (11, 12, 14, 19). Twenty-nine studies (5, 12, 15–17, 28–32, 34, 36) utilized 1.5-T MRI, five studies (13, 14, 19, 33, 35) used 3.0-T MRI, and one study used 1.5-T and/or 3.0-T MRI (26). Half of the included studies were from Asian countries (5, 11, 12, 16, 19, 30, 33–35). To diagnose NME lesions, seven studies utilized pathology or clinical diagnostic criteria follow-up (5, 12, 17, 28, 31, 32, 35), while 11 studies used pathology as a reference standard (11, 13–16, 19, 29, 30, 33, 34, 36).

**Table 1A:** The baseline characteristics of the included studies.

Author (year of publication)	Country	Magnet field strength, manufacturer	Reference	Data source	b value (s/mm^2^)	Study design	Diagnostic criteria	Enhanced scan sequence and direction	Slice thickness	Position of the patients
Akiko Shimauchi 2015 (5)	Japan	1.5 T, Siemens	Histopathology or follow-up	Single institution	–	Retrospective	IEP; distribution; IEP and distribution	3D T1 fs VIBE, coronal	0.9 mm	–
Andréa Alves Maciel Di Ninno 2021 (28)	Brazil	1.5 T, Siemens/GE	Histopathology and follow-up	Bi-center	–	Prospective	Distribution	3D fs T1 fast spoiled gradient-echo, sagittal	–	Prone
Eduardo de Faria Castro Fleury 2022 (29)	Brazil	1.5 T, Siemens	Histopathology	Single institution	–	Retrospective	IEP; distribution	fs T1-GE, sagittal	–	Prone
Fatma Zeinhom Moukhtar 2014 (15)	Egypt	1.5 T, GE	Histopathology	Single institution	750	Retrospective	ADC threshold 1.35	3D VIBRANT, axial	1.2 mm	Prone
Gang Liu 2022 (30)	China	1.5 T, Philips	Histopathology	Single institution	1,000	Retrospective	IEP; distribution; TIC; ADC threshold 1.3	3D T1-FFE, coronal	2 mm	Prone
Hale aydIn 2019 (31)	Turkey	1.5 T, GE	Histopathology or follow-up	Single institution	–	Retrospective	IEP; distribution; diffusion restriction; TIC; IEP and distribution	2D T1 fs GE, axial	2.8 mm	Prone
Hidetake Yabuuchi 2010 (11)	Japan	1.5 T, Philips	Histopathology	Single institution	–	Retrospective	IEP; distribution; TIC; ADC threshold; BI-RADS	3D T1-FFE with WATS, coronal	1 mm	Prone
Isabelle Thomassin-Naggara 2011 (32)	Canada	1.5 T, Philips	Histopathology or follow-up	Single institution	–	Retrospective	IEP; distribution; TIC	fs T1-GE, axial	1 mm/1.6 mm	Prone
Jiejie Zhou 2021 (33)	China	3.0 T, GE	Histopathology	Single institution	–	Retrospective	IEP; distribution; BI-RADS; IEP and distribution; DL; ML	3D VIBRANT	1.2 mm	–
K. Pinker 2013 (13)	Austria	3.0 T, Siemens	Histopathology	Single institution	–	Prospective	IEP; distribution; TIC	3D T1-FLASH, coronal; T1-VIBE	–	Prone
Keiichi Sotome 2007 (34)	Japan	1.5 T, GE	Histopathology	Single institution	–	Retrospective	Distribution	2D fs T1-SPGR, axial	5 mm	–
Lijun Wang 2022 (35)	China	3.0 T, GE	Histopathology or follow-up	Single institution	–	Retrospective	DL; distribution; BI-RADS	–	–	Prone
Liuquan Cheng 2013 (16)	China	1.5 T, GE	Histopathology	Single institution	1,000	Retrospective	ADC threshold 1.35	3D VIBRANT, axial	1.2 mm	Prone
Magdalena Lunkiewicz 2020 (36)	Switzerland	1.5 T, Siemens/3.0 T, Siemens	Histopathology	Two institutions	–	Retrospective	IEP; distribution	T1 fat-saturated, axial	1 mm	Prone
Maria Adele Marino 2022 (14)	USA/Italy	3.0 T, Siemens	Histopathology	Single institution	850	Retrospective	IEP; distribution; TIC; ADC threshold 1.305; DCE; DCE+ADC	T1 VIBE, coronal	–	Prone
Sibel Kul 2014 (17)	Turkey	1.5 T, Siemens	Histopathology or follow-up	Single institution	1,000	Retrospective	ADC threshold 0.9	3D fs T1 FLASH	1.5 mm	Prone
Tsugumi Imamura 2010 (12)	Japan	1.5 T, Philips	Histopathology or follow-up	Single institution	1,000	Retrospective	IEP; distribution; TIC; ADC threshold 1.1	3D fs T1-FFE, coronal	2 mm	Supine
Xiaoping Yang 2020 (19)	China	3.0 T, Siemens	Histopathology	Single institution	800	Retrospective	Kinetic curve plateau or washout; ADC threshold 1.235	d T1-weighted gradient echo fast low-angle shot	1.6 mm	Prone
								(FLASH) image with fat suppression (FS-CE-T1W-GRE)		

GE, gradient echo; FFE, fast field echo; VIBRANT, volume imaging for breast assessment; 3D fs T1-SPGR, coronal three-dimensional spoiled gradient-recalled echo pulse; THRIVE, T1-weighted high-resolution isotropic volume examination; FLASH, coronal T1-weighted turbo fast low-angle shot; VIBE T1-weighted volume-interpolated breath-hold examination; FSPGR, fast spoiled gradient-recalled echo.

**Table 1B:** The baseline characteristics of the included studies.

Author (year of publication)	Study design	Sample inclusion time	Age	Premenopausal	Cancer prevalence	DCIS	IDC with DCIS	IDC	ILC	Mucinous carcinoma	Apocrine carcinoma
Akiko Shimauchi 2015 (5)	Retrospective	2009.4–2010.12	49 (29–80)	–	57.3% (86/150)	59.3% (51/86)	–	25.6% (22/96)	3.49% (3/86)	2.33% (2/86)	–
Andréa Alves Maciel Di Ninno 2021 (28)	Prospective	2011.1–2015.7	–	–	61.5% (48/78)	57.9% (22/38)	21.1% (8/53)	15.8% (6/38)	2.6% (1/38)	–	–
Eduardo de Faria Castro Fleury 2022 (29)	Retrospective	2018.1–2021.7	49.3	–	6.25% (6/96)	83.3% (5/6)	–	16.7 (1/6)	–	–	–
Fatma Zeinhom Moukhtar 2014 (15)	Retrospective	2012.7–2013.5	–	–	69.2% (27/39)	77.8% (21/27)	–	22.2% (6/27)	–	–	–
Gang Liu 2022 (30)	Retrospective	2018.3–2021.3	-, (18–70)	–	47.5% (56/118)	57.1% (32/56)	–	42.9% (24/56)	–	–	–
Hale aydIn 2019 (31)	Retrospective	2015.1–2017.12	45.9 (18–79)	–	23.3% (30/129)	–	–	–	–	–	–
Hidetake Yabuuchi 2010 (11)	Retrospective	2006.4–2007.11	55.4 (33–82)	–	68.9% (31/45)	54.8% (17/31)	–	41.9% (13/31)	–	–	3.2%(1/31)
Isabelle Thomassin-Naggara 2011 (32)	Retrospective	2008.1–2009.6	51.4 (28–78)	43.2% (19/44)	47.7% (21/44)	27.3% (12/21)	19.0% (4/21)	6.8% (3/21)	4.5% (2/21)	–	–
Jiejie Zhou 2021 (33)	Retrospective	2017.1–2019.12	–	–	69.3% (104/150)71.1% (32/45)	42.3% (44/104)	–	53.8% (56/104)	–	–	–
K. Pinker 2013 (13)	Prospective	2007.9–2011.9	–	–	47.2% (17/36)	4.8% (14/209)	–	51.7% (152/209)	9.2% (27/209)	2.4% (7/209)	–
Keiichi Sotome 2007 (34)	Retrospective	2003.5–2005.5	49.4 (24–75)	–	56.3% (18/32)	16.7% (3/18)	–	16.7% (3/18)	38.9% (7/18)	–	–
Lijun Wang 2022 (35)	Retrospective	2014.1–2015.9	48 (40–57)	–	32.2% (68/211)	–	–	–	–	–	–
Liuquan Cheng 2013 (16)	Retrospective	2009.7–2010.5	–	–	68.9% (42/61)	33.3% (14/42)	–	66.7% (28/42)	–	–	–
Magdalena Lunkiewicz 2020 (36)	Retrospective	2011.1–2017.5	52.6 (31.5–91)	–	26.4 (19/72)	31.6% (6/19)	5.3% (1/19)	26.3% (5/19)	15.8% (3/19)	–	–
Maria Adele Marino 2022 (14)	Retrospective	2007.9–2013.7	51.8 (26–76)	–	59.1% (39/66)	10.3% (4/39)	–	61.5% (24/39)	23.1% (9/39)	–	–
Sibel Kul 2014 (17)	Retrospective	2008.8–2012.11	–	–	38.4% (28/73)	–	–	–	–	–	–
Tsugumi Imamura 2010 (12)	Retrospective	2005.8–2006.9	51.5 (27–81)	–	59.26% (16/27)	18.8% (3/16)	62.5% (10/16)	1.23% (2/16)	–	–	–
Xiaoping Yang 2020 (19)	Retrospective	2014.1–2018.9	44.41 ± 10.64 and 45.56 ± 11.96	–	77.19% (281/364)	–	–	–	–	–	–

DCIS, ductal carcinoma *in situ*; IDC, invasive ductal carcinoma; ILC, invasive lobular carcinoma.

### Quality assessment

**Figure 2:**
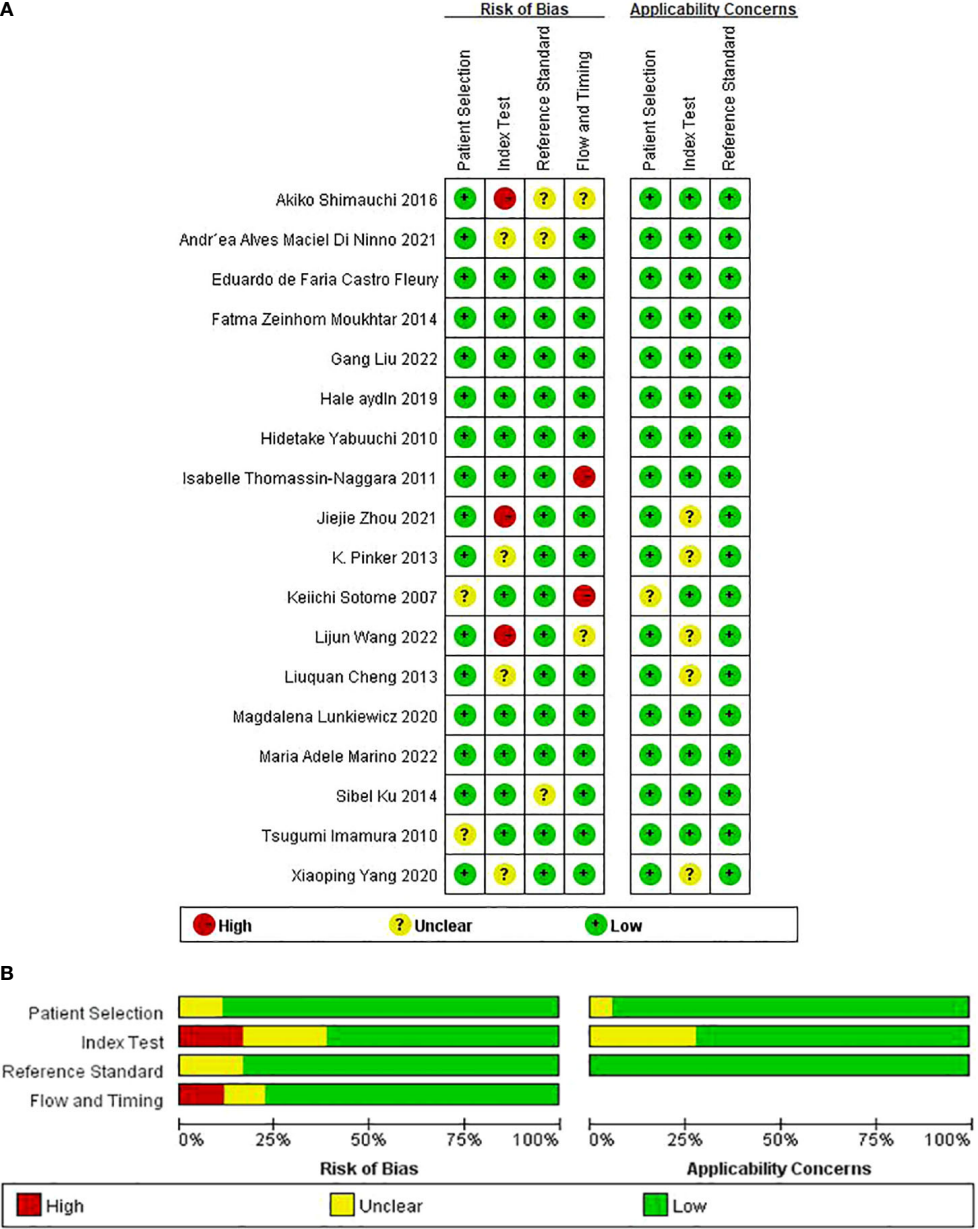
Methodological quality summary of all included studies by using QUADAS-2 **(A, B)**.

The results of the QUADAS-2 assessment are compiled in **Figures 2A, B**. Of the studies, 11.1% (2/18) in the patient selection domain were scored as “unclear” (12, 34). Two studies did not report consecutive patients. Index test result blinding when interpreting the reference standard was unclear in four studies (13, 16, 19, 28). Index test result blinding when interpreting the reference standard was unclear in three studies (5, 33, 35). Three investigations did not report whether the threshold was pre-specified (5, 17, 28). Because there was inconsistent histopathological analysis used as the reference standard for all included cases, two studies were classified as having unclear risk of flow and timing bias domain (5, 35).

### Meta-analysis

#### Pooled sensitivity and specificity analysis of DCE-MRI, DWI-MR, and combination models

For DCE-MRI (38 datasets in 13 studies), sensitivity and specificity varied considerably across individual studies (0.33–0.96 of sensitivity and 0.33–0.95 of specificity), with meta-analytic summary sensitivity and specificity 95% CI of 0.58 (0.50, 0.66) and 0.72 (0.64, 0.78), respectively (**Table 2**).

**Table 2:** Results of pooled estimates and heterogeneity measures for MRI studies of the NME lesions.

	No. of studies	No. of data	No. of lesions	Sensitivity (95% CI)	Heterogeneity		Specificity (95% CI)	Heterogeneity		AUC	Heterogeneity	
					Cochran Q *p-*value	I^2^ (%)		Cochran Q *p*-value	I^2^ (%)		Cochran Q *p*-value	I^2^ (%)
DCE	13	38	4,223	0.58 (0.50, 0.66)	<0.01	0.9069	0.72 (0.64, 0.78)	<0.01	0.9036	0.70 (0.66–0.74)	<0.01	0.9961
DWI	9	9	844	0.84 (0.77, 0.89)	<0.01	0.661	0.69 (0.59, 0.78)	<0.01	0.7674	0.84 (0.81–0.87)	<0.01	0.9299
Combination model	4	4	193	0.88 (0.78, 0.94)	0.12	0.4859	0.79 (0.63, 0.89)	<0.01	0.4926	0.90 (0.88–0.93)	0.258	0

NME, non-mass enhancement; DCE, dynamic contrast enhancement; DWI, diffusion-weighted imaging; AUC, area under the curve.

For DWI-MRI (nine datasets in nine studies), sensitivity and specificity demonstrated relatively small degrees of variation across individual studies (0.69–0.94 of sensitivity and 0.48–0.81 of specificity). The meta-analytic summary sensitivity and specificity were 0.84 (0.77, 0.89) and 0.69 (0.59, 0.78), respectively.

**Figure 3:**
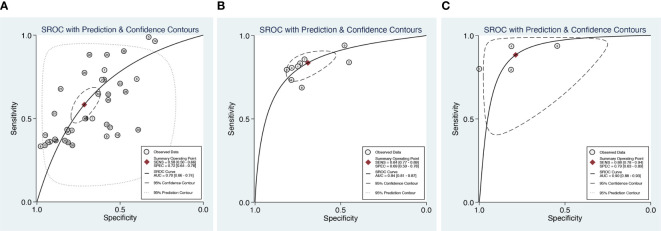
Summary receiver operating characteristic (SROC) curves of DCE **(A)**, DWI **(B)**, and combination model **(C)** with 95% confidence intervals.

For DWI combined with DCE (four datasets in four studies), sensitivity and specificity demonstrated relatively small degrees of variation across individual studies (0.79–0.94 of sensitivity and 0.55–1 of specificity). The meta-analytic summary sensitivity and specificity were 0.88 (0.78, 0.94) and 0.79 (0.63, 0.89), respectively. **Figure 3** depicts the hierarchical ROC curves of the region-based DCE-MRI, DWI-MRI, and combination model studies. The sensitivity and specificity ROC curves did not demonstrate a threshold effect.

**Figure 4:**
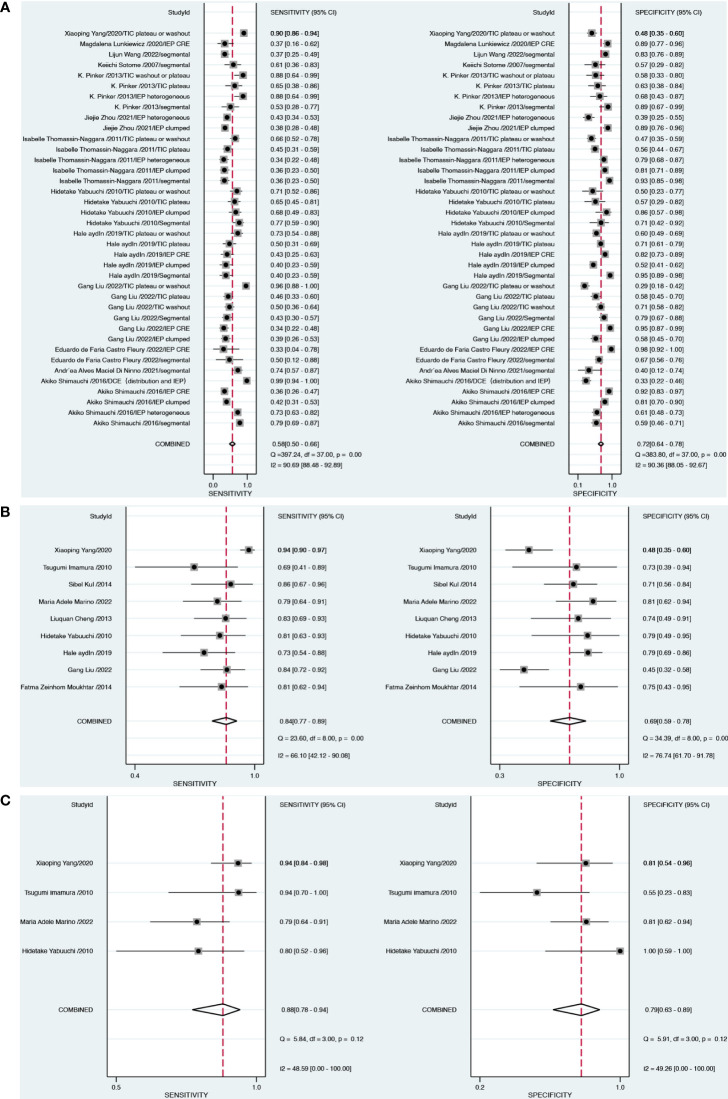
Forest plots demonstrate the pooled sensitivity and specificity of DCE **(A)**, DWI **(B)** and combination model **(C)**.

Heterogeneity testing demonstrated that there was considerable heterogeneity among the studies for DCE and specificity of DWI alone (I^2^ > 75%). There was no significant heterogeneity (I^2^ < 50%) in DCE combined with DWI approaches. The forest plots of all the studies are shown in **Figure 4**, along with the I^2^ score and pooled estimates for each modality.

#### Subgroup analysis

Based on the DCE and DWI of malignant and benign NME lesions, we performed a subgroup analysis. A subgroup analysis showed the DCE covariates, including segmental, heterogeneous, clumped, CRE, washout, plateau, and washout/plateau features. Distribution features produced the best diagnostic performance for separating malignant from benign NME lesions with an AUC 95% CI of 0.72 (0.68–0.75), as **Table 3** illustrates. Additionally, washout/plateau had the highest pooled sensitivity of 0.84 (0.71, 0.92), while CRE had the highest pooled specificity of 0.92 (0.86, 0.96). DWI subgroup analysis showed that an ADC mean threshold <1.3 × 10^−3^ mm^2^/s had a slightly higher sensitivity of 0.86 (0.74, 0.93) than 0.82 (0.75, 0.87) with ADC ≥ 1.3 × 10^−3^ mm^2^/s and similar specificity.

**Table 3:** Summary of subgroup analyses: breakdown of malignant and benign NME lesions.

Parameter	No. of studies	No. of data	No. of lesions	Sensitivity (95% CI)	Heterogeneity		Specificity (95% CI)	Heterogeneity		AUC	Heterogeneity	
					Cochran Q *p-*value	I^2^ (%)		Cochran Q *p-*value	I^2^ (%)		Cochran Q *p-*value	I^2^ (%)
Distribution	10	10	996	0.56 (0.44, 0.68)	<0.01	0.8385	0.77 (0.66, 0.86)	<0.01	0.8698	0.72 (0.68–0.75)	<0.01	0.9732
Heterogeneous	4	4	467	0.60 (0.36, 0.80)	<0.01	0.9198	0.63 (0.46, 0.77)	<0.01	0.8517	0.65 (0.61–0.69)	<0.01	0.8929
Clumped	6	6	723	0.42 (0.35, 0.48)	0.06	0.5289	0.76 (0.62, 0.86)	<0.01	0.8901	0.49 (0.44–0.53)	0.004	0.7976
CRE	5	5	565	0.36 (0.30, 0.44)	0.94	0	0.92 (0.86, 0.96)	<0.01	0.7609	0.47 (0.43–0.52)	0.015	0.713
Washout	5	5	459	0.23 (0.12, 0.38)	<0.01	0.8208	0.89 (0.79, 0.94)	0.01	0.7388	0.66 (0.62–0.70)	<0.01	0.9089
Plateau	5	5	459	0.52 (0.44, 0.60)	0.29	0.1965	0.63 (0.55, 0.69)	0.3	0.1861	0.58 (0.54–0.63)	0.493	0
Washout/plateau	6	6	745	0.84 (0.71, 0.92)	<0.01	0.8513	0.48 (0.39, 0.57)	0.01	0.6699	0.62 (0.58–0.67)	<0.01	0.9049
ADC cut-off ≥1.3	4	4	284	0.82 (0.75, 0.87)	0.94	0	0.68 (0.50, 0.82)	<0.01	0.7871	0.83 (0.80–0.86)	0.031	0.6409
ADC cut-off <1.3	4	4	431	0.86 (0.74, 0.93)	<0.01	0.8154	0.67 (0.52, 0.80)	0.03	0.6766	0.84 (0.80–0.87)	0.001	0.8288

NME, non-mass enhancement; AUC, area under the curve; CRE, clustered ring enhancement; ADC, apparent diffusion coefficient.

#### Meta-regression analysis

The DCE and DWI-MRI underwent meta-regression analysis to identify the source of heterogeneity. The meta-regression results are shown in **Table 4**. For the DCE model, the pooled specificity of studies diagnostic criteria with distribution or TICs 0.74 (0.66–0.83) was higher than that of studies with IEP as diagnostic criteria 0.68 (0.57–0.78). Asians had a higher pooled sensitivity of 0.66 (0.57–0.75) as compared to Caucasians, which had 0.45 (0.33–0.58).

**Table 4:** Univariable meta-regression evaluating the effect of confounding factors on sensitivity and specificity of DCE and ADC.

Parameter	Category	No. of data	Sensitivity (95% CI)	*p*	Specificity (95% CI)	*p*
DCE						
Predesign	Prospective	6	0.49 (0.09–0.89)	0.64	0.85 (0.67–1.00)	0.58
	Retrospective	41	0.59 (0.51–0.67)		0.70 (0.63–0.77)	
Publication year	After 2013	37	0.59 (0.50–0.68)	0.82	0.71 (0.63–0.79)	0.11
	Before 2013	10	0.56 (0.39–0.74)		0.73 (0.60–0.87)	
Diagnostic criteria	Single IEP	19	0.58 (0.45–0.70)	0.45	0.68 (0.57–0.78)	0.02
	Others	28	0.59 (0.48–0.70)		0.74 (0.66–0.83)	
MR magnet strength	Only 3.0 T	8	0.91 (0.75–1.00)	0.05	0.48 (0.01–0.96)	0.3
	Others	39	0.57 (0.49–0.65)		0.72 (0.65–0.79)	
Reference	Histopathology	26	0.59 (0.47–0.71)	0.59	0.72 (0.62–0.82)	0.09
	Histopathology or follow-up	21	0.58 (0.46–0.69)		0.71 (0.62–0.81)	
Cancer prevalence	≥50%	21	0.64 (0.53–0.75)	0.78	0.71 (0.61–0.81)	0.05
	<50%	26	0.53 (0.41–0.64)		0.72 (0.63–0.81)	
Race	Caucasian	21	0.45 (0.33–0.58)	0.02	0.76 (0.67–0.85)	0.31
	Asian	26	0.66 (0.57–0.75)		0.68 (0.58–0.77)	
ADC						
Publication year	After 2013	7	0.85 (0.79–0.91)	0.57	0.67 (0.57–0.77)	0.25
	Before 2013	2	0.77 (0.60–0.93)		0.77 (0.56–0.98)	
Diagnostic criteria	ADC cut-off ≥1.3 × 10^−3^ mm^2^/s	4	0.82 (0.74–0.91)	0.01	0.66 (0.51–0.81)	0.18
	ADC cut-off <1.3 × 10^−3^ mm^2^/s or not reported	5	0.85 (0.77–0.92)		0.71 (0.59–0.83)	
MR magnet strength	3.0 T	2	0.90 (0.84–0.96)	0.17	0.65 (0.45–0.86)	0.33
	1.5 T	7	0.80 (0.74–0.87)		0.70 (0.59–0.81)	
Reference	Histopathology	6	0.86 (0.80–0.91)	0.3	0.65 (0.53–0.77)	0.04
	Histopathology or follow-up	3	0.77 (0.65–0.90)		0.76 (0.64–0.88)	
Cancer prevalence	≥50%	6	0.85 (0.78–0.91)	0.12	0.73 (0.61–0.85)	0.69
	<50%	3	0.81 (0.70–0.92)		0.66 (0.50–0.82)	
Max b value	≥800 s/mm^2^ *vs.*	7	0.85 (0.79–0.91)	0.42	0.66 (0.55–0.77)	0.07
	<800 s/mm^2^ or not reported	2	0.78 (0.63–0.92)		0.77 (0.62–0.92)	
Race	Caucasian	4	0.80 (0.71–0.90)	0	0.77 (0.68–0.85)	0.86
	Asian	5	0.86 (0.79–0.92)		0.58 (0.47–0.70)	

DCE, dynamic contrast enhancement; ADC, apparent diffusion coefficient.

For the DWI MRI, the specificity of studies using histopathology or follow-up as a reference standard with 0.76 (0.64–0.88) was higher than that of studies using histopathology as a reference with a specificity of 0.65 (0.53–0.77). Using the threshold of ADC < 1.3 × 10^−3^ mm^2^/s, the sensitivity and specificity of 0.85 (0.77–0.92) and 0.71 (0.59–0.83), respectively, were higher than 0.82 (0.77–0.92) and 0.66 (0.51–0.81), respectively, with ADC ≥ 1.3 × 10^−3^ mm^2^/s as cut-off. Likewise, Asians had a higher pooled sensitivity of 0.86 (0.79–0.92) as compared to Caucasians, which had 0.80 (0.71–0.90). As the number of studies is limited, we did not evaluate meta-regression analysis for the combination model.

### Publication bias

**Figure 5:**
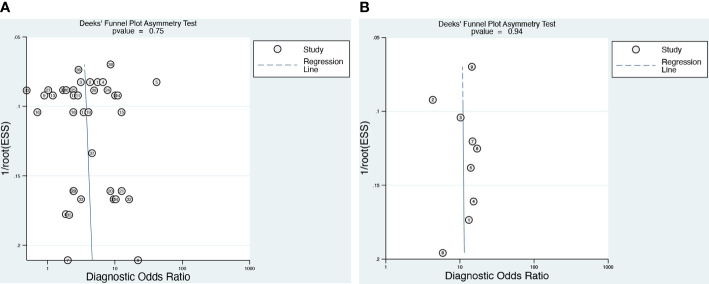
The funnel plot of publication bias for DCE **(A)** and DWI **(B)**.

Deeks’ funnel plot (**Figure 5**) of the DCE-MRI and DWI-MR demonstrated that there was no publication bias (*p* = 0.75 and 0.94, respectively).

### Clinical utility

**Figure 6:**
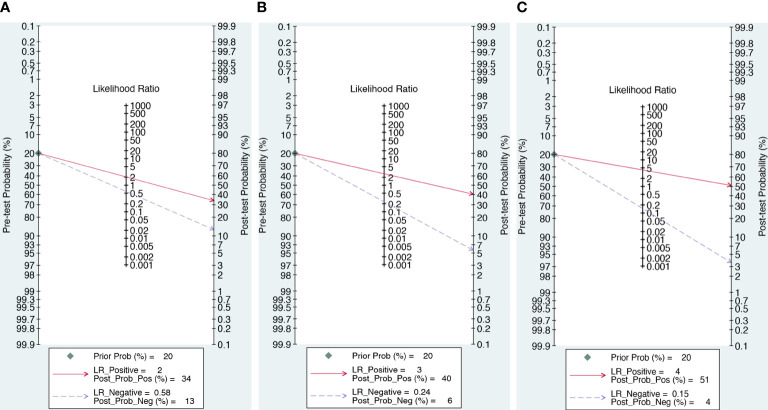
The posttest probabilities on Fagan plots for the DWI **(A)**, DWI **(B)** and combination model **(C)**.

As illustrated in **Figure 6**, we computed the posttest probabilities on Fagan plots for the DWI, DCE alone, and DCE combined with DWI models. In the event of a positive pretest, using the DCE combined with DWI model would increase the posttest probability to 51 from 20% with a positive likelihood ratio (PLR) of 4, while in the event of a negative pretest, it would decrease to 4% with a negative likelihood ratio (NLR) of 0.15.

## Discussion

In the present meta-analysis, we investigated the diagnostic value of the combination model (DCE and DWI), DCE alone, and DWI alone in NME breast lesions. We demonstrated that combining DCE and DWI may improve sensitivity and specificity when compared to either DCE or DWI alone. We first systematically assessed the ability of DWI to differentiate between malignant and benign NME lesions, and we discovered that DWI outperformed DCE in terms of diagnostic accuracy and had a comparatively high pooled sensitivity. The findings confirmed the possibility of DWI as a shorter and simpler protocol for breast MRI.

DCE-MRI is a vital technique for identifying NME lesions, as well as internal enhancement models and morphologic characteristics, and is the most important parameter (19). We found that DCE-MRI had moderate sensitivity and specificity of 0.58 and 0.72, respectively, which were similar to the 2013 meta-analysis of Shao et al. (18), who reported that the pooled weighted estimates of sensitivity and specificity were 0.5, and 0.8, respectively. One possible explanation for moderate sensitivity may be that there was no association between malignancy and some of the included DCE features, such as clumped pattern enhancement and homogeneity, distribution type, and clumped pattern combinations (31). Likewise, various studies have found no connection between homogeneous patterns and malignancy (37, 38). Therefore, we used subgroup analysis to find the valuable DCE features.

The subgroup results revealed that the washout/plateau TICs demonstrated the highest diagnostic sensitivity, while the CRE characteristic had the highest diagnostic specificity. CRE is the main feature of intraductal carcinoma of NME, accounting for 72.2% of all malignant lesions in this study, and five studies were not reported. The pathological basis of CRE in intraductal carcinoma is its abundant blood supply. After enhancement, the matrix around the catheter and the guide tube wall can be improved, and small ring enhancement can be demonstrated when perpendicular to the catheter section (31). When lesions exhibit CRE features, they should be diagnosed as malignant.

Research has shown that TIC is useful in the diagnosis of breast disease (11, 13, 19, 30–32). This finding may be attributed to the presence of varying degrees of arteriovenous shunts in breast tissue, as well as capillary hypertrophy and high endothelial permeability in malignant breast lesions (31, 32). Our findings indicate that plateau curves or washout TICs are highly sensitive but have low specificity in diagnosing NME BCa. Unfortunately, TICs are semi-quantitative analyses, and plateau curves might indicate either malignant or benign lesions, reducing TICs’ diagnostic ability.

Furthermore, it is worth noting that the dynamic type is related to the pathological type of breast cancer. Dynamic curves can help distinguish between benign lesions and invasive NME BCa, but they cannot tell the difference between DCIS and benign lesions. Moreover, a washout curve could help differentiate DCIS from invasive NME lesions (31). We were unable to define the pathology type due to the small sample size, and more research is still needed to determine how curve type and pathological type are related.

DWI combined with the quantitative ADC value has been useful in the diagnosis of breast lesions (16, 19, 30). In this investigation, we discovered that DWI and ADC values can help diagnose malignant NME lesions. It appears to have increased sensitivity to NME BCa. However, we cannot recommend ADC thresholds for NME lesions because the ADC values of included studies ranged from 0.9 to 1.35 × 10^−3^ mm^2^/s. Moreover, subgroup results indicated that ADC values with a mean threshold of <1.3 × 10^−3^ mm^2^/s had slightly higher sensitivity than those with a threshold of ≥1.3 × 10^−3^ mm^2^/s. Similar to our results, previous studies have found an ADC mean threshold of <1.3 × 10^−3^ mm^2^/s, which is considered a suspicious diffusion hindrance level by the EUSOBI DWI working group consensus (39). Unfortunately, more research was not conducted to determine the optimal individual threshold of ADC value for differentiating between benign and malignant NME lesions due to the small sample size and variability.

Our findings revealed that the DWI combined with DCE model outperformed the DCE- and DWI-alone models in discriminating between benign and malignant NME. This result highlights the importance of actively integrating DWI with the morphologic and functional data from DCE-MRI.

In our experience, these sequences often complement one another, thereby reducing erroneous readings. Moreover, breast DCE combined with DWI can provide unique morphological, functional, and molecular information about breast tumors, which may significantly improve the BCa diagnostic level. Additionally, DWI is fast and sensitive, but its spatial resolution is poor, and it cannot observe lesions comprehensively (16). Therefore, it is better to combine it with DCE, which has high spatial resolution.

However, the specificity and sensitivity of included studies range from one another, which can be explained by a variety of reasons such as variances in research group size, use of different BI-RADS lexicons resulting in distinct internal enhancement classifications, and changes in evaluation. Heterogeneity is common in meta-analyses. In our study, the different diagnostic criteria significantly influenced heterogeneity in DCE-MRI data. The meta-regression analysis revealed that the DCE model had higher diagnostic sensitivity and specificity in segmental or TIC than in IEP. A previous review reported that IEP clumped pattern enhancement and homogeneity are common in benign lesions (31). As a result, the specificity of studies may be limited. Furthermore, study design can impact research quality and lead to heterogeneity.

In the DWI model, the different reference standard was the source of heterogeneity. There is higher sensitivity in reference to histopathology than histopathology or follow-up, but the specificity was limited. Because the accuracy of the diagnostic test is determined by comparing the results of the index test with those of the reference standard, comprehension of the reference standard may influence the interpretation of the index test findings. However, excluding some benign NME lesions that reference standards based on follow-up, this maybe lead to sample selection bias. In addition, different imaging parameters also create heterogeneity.

This is the first meta-analysis with adequate MRI data to evaluate DWI, DCE methods, and the current combination model for assessing NME lesions. Furthermore, Deek’s funnel plot demonstrated the absence of published bias, implying that our findings are reliable. However, there are certain limitations. First, there was significant heterogeneity for both sensitivity and specificity. As a result, we conducted subgroup analysis and meta-regression analysis to investigate the sources of study heterogeneity, as described above. Second, there may have been interpretation bias because six of the included studies used reference criteria ranging from pathological analysis to surgical or biopsy findings to radiological follow-up. Third, the acquisition of various indicators is dependent on multiple imaging parameters (b value, Tesla), which resulted in some variation between investigations. Furthermore, differences in MR sequence input, ground truth, and other variables may influence the results, although this is considered a minor limitation, as most studies had similar source data. To address these limitations, we recommend further large-scale studies to unify the use of b values and DCE parameters in diagnosing NME lesions.

In conclusion, our findings showed that the combination model (DCE and DWI) provided extremely good diagnostic performance in distinguishing between malignant and benign NME lesions. DWI has a substantially higher sensitivity for detecting NME lesions than DCE. The DCE-CRE feature was the most specific test for detecting NME cancers. Therefore, further study should combine DWI with DCE to test the accuracy of differentiating cancers from NME benign lesions.

## Data availability statement

The original contributions presented in the study are included in the article/supplementary material. Further inquiries can be directed to the corresponding author.

## Author contributions

JZ: Software, Writing – original draft. LL: Conceptualization, Investigation, Methodology, Writing – original draft. LZ: Methodology, Supervision, Writing – review & editing. XZ: Data curation, Investigation, Writing – original draft. MT: Formal Analysis, Validation, Writing – review & editing. XL: Methodology, Supervision, Writing – review & editing. XLZ: Formal Analysis, Supervision, Writing – review & editing.
